# Exploring experiences of Faculty of Health Sciences students on interprofessional collaboration in the healthcare settings in the Khomas region, Namibia

**DOI:** 10.4102/hsag.v31i0.3399

**Published:** 2026-07-24

**Authors:** Daniel O. Ashipala, Rossaline Mlunga, Suama Kuugongelwa

**Affiliations:** 1 Department of General Nursing Sciences, Faculty of Health Sciences and Veterinary Medicine, University of Namibia, Rundu, Namibia; 2 Department of General Nursing Sciences, Faculty of Health Sciences and Veterinary Medicine, University of Namibia, Windhoek, Namibia

**Keywords:** experiences, health sciences students, interprofessional collaboration, Namibia healthcare, patient care, teamwork

## Abstract

**Background:**

Globally, interprofessional collaboration (IPC) has been shown to improve patient outcomes and foster teamwork. While IPC forms an integral part of healthcare delivery, enabling professionals from various disciplines to work together in achieving holistic patient care, in Namibia, there is little research on the experiences of Faculty of Health Sciences students on IPC.

**Aim:**

The aim of this study was to explore and describe the experiences of Faculty of Health Sciences students on IPC in healthcare settings within the Khomas Region, Namibia.

**Setting:**

This study was conducted at the National University in Namibia.

**Methods:**

A qualitative exploratory contextual design was used. Data were collected through individual, in-depth, semi-structured interviews with 16 Faculty of Health Sciences students. All interviews were audio recorded with a digital voice recorder followed by verbatim transcriptions, with the participants’ permission. The collected data were analysed thematically to identify recurring themes.

**Results:**

Three main themes emerged: (1) Understanding IPC as a learning and teamwork opportunity, (2) barriers to ineffective IPC in clinical settings and (3) mechanisms for enhancing IPC in healthcare settings.

**Conclusion:**

This study revealed that IPC is seen as a pathway to better teamwork and improved patient care outcomes among healthcare students. However, addressing challenges such as fostering effective interprofessional relationships is crucial for optimising IPC in healthcare settings.

**Contribution:**

Understanding student’s experiences on IPC can contribute to the development of ongoing strategies and targeted interventions geared towards enhancing strong IPC and promoting teamwork, effective communication and improved patient-centred care.

## Introduction

Interprofessional collaboration (IPC) is fundamental to delivering high-quality, patient-centred healthcare, as it fosters teamwork among professionals from diverse disciplines to address patient needs holistically (Schot, Tummers & Noordegraaf 2020). The World Health Organization (WHO) emphasises IPC as a critical practice for improving global healthcare outcomes, highlighting its role in reducing medical errors, enhancing efficiency and ensuring better patient experiences (Moghaddasi 2019). Studies from high-income countries demonstrate that integrating IPC into healthcare systems leads to improved clinical decision-making and better health outcomes (Reeves et al. 2010). Consequently, IPC has become a core competency in healthcare education, with growing emphasis on training future professionals to collaborate effectively in multidisciplinary teams. However, despite its proven benefits, IPC implementation varies across regions, particularly in resource-limited settings where structural and systemic barriers persist (Dow & Reeves 2016).

Interprofessional collaboration has been widely integrated into the curricula of developed countries (high-income countries – HICs). While its integration in low- and middle-income countries (LMICs) is increasing but remains largely in the early, emerging or pilot stages (Neill et al. 2023). While HICs focus on strengthening established, often mandatory IPE programmes to manage chronic conditions and patient safety, LMIC implementation is frequently limited by resource constraints, rigid hierarchical structures and lack of faculty training (Sunguya et al. 2014).

In daily practice, nurses frequently find themselves in coordinating roles that are often unrecognised formally.

Their continuous presence at the bedside allows them to detect subtle changes in patient conditions that may go unnoticed by others. Nurses communicate patient concerns to physicians, clarify care instructions for families and liaise with allied health professionals to ensure continuity and safety. Baek et al. (2023) emphasises that nurses often serve as intermediaries within healthcare teams, facilitating information flow and supporting decision-making processes. While this intermediary role is not always visible in organisational hierarchies, it is essential to the smooth functioning of interprofessional teams. Nurses’ engagement in collaboration directly influences the integration of care and the efficiency of patient management systems.

While interprofessional education has gained global recognition, its integration into local professional development structures varies. Exposure to collaborative training during formal education may not adequately prepare nurses for navigating complex institutional cultures. Without continuous professional development opportunities focused specifically on teamwork, conflict resolution and shared leadership, IPC remains dependent on individual initiative rather than on systematic reinforcement (Bester, Van Wyk & Maree 2024).

Therefore, such differences in disciplinary training backgrounds can further influence expectations regarding authority and participation. When professions are socialised differently in their decision-making processes, alignment requires deliberate effort.

To address these challenges, educational strategies such as interprofessional education (IPE) have been implemented in healthcare training programmes. Interprofessional education aims to expose students from diverse professional backgrounds to shared learning experiences, fostering early appreciation of mutual respect, role understanding and collaborative competencies (Uwimana et al. 2025). While the theoretical benefits of IPE are clear, the practical impact on long-term professional behaviour remains uncertain. Clinical environments frequently reshape ideals encountered during training, reinforcing hierarchical norms and limiting opportunities for meaningful engagement Bsharat et al. (2025). In this way, students may learn the value of collaboration in theory, but the realities of staffing shortages, high workloads and entrenched authority structures can inhibit its consistent practice.

Extensive research in high-income countries has demonstrated that embedding IPC within healthcare systems leads to improved clinical decision-making, enhanced patient safety and higher healthcare efficiency. In the United States and Canada, IPC has a standardised component of healthcare education and practice, with IPC models being widely adopted in hospitals and primary care settings (Reeves et al. 2010). Studies from these countries have shown that collaborative care models improve patient recovery rates, reduce hospital readmissions and enhance overall healthcare delivery (Hilty et al. 2015). Beyond routine healthcare, IPC plays a pivotal role in responding to global health crises. During the coronavirus disease 2019 (COVID-19) pandemic, countries that implemented strong IPC frameworks were able to coordinate multidisciplinary responses effectively, ensuring rapid patient assessment, efficient use of medical resources and improved infection control measures (Howe 2022). In contrast, nations with fragmented healthcare systems and weak IPC struggled to manage patient surges, leading to higher mortality rates and systemic inefficiencies (Doherty 2020).

Despite its proven benefits, IPC implementation varies significantly across regions, with LMICs facing greater challenges in integrating interprofessional approaches into healthcare systems (Edoh 2021). Structural and systemic barriers, such as limited financial resources, workforce shortages, lack of standarised IPC training and deeply rooted professional hierarchies, hinder the widespread adoption of collaborative healthcare models (Dow & Reeves 2016). These disparities highlight the need for context-specific strategies to strengthen IPC, particularly in resource-limited settings like sub-Saharan Africa, where effective interprofessional teamwork could significantly enhance healthcare outcomes (Pajalich 2019).

The University of Namibia (UNAM), the country’s leading institution for training health professionals in disciplines such as nursing, medicine, pharmacy and allied health sciences, serves as an education hub in Namibia. As part of their education, students undertake clinical placements in hospitals, clinics and community health centres, providing them with opportunities to engage in real-world IPC. For health sciences students, clinical placements serve as a critical period for developing collaborative competencies. These placements provide an opportunity to work alongside professionals from other disciplines, learn about their roles and observe the dynamics of team-based care in action. However, without structured support and intentional efforts to promote IPC during these placements, students may struggle to navigate interprofessional interactions. They may also perpetuate the silos and hierarchies that have historically hindered collaboration in healthcare settings.

Interprofessional education was introduced at the UNAM by integrating community-centred, collaborative curriculum models across all the schools within the faculty reinforced by international partnerships and advocacy from the Africa Interprofessional Education Network (AfrIPEN 2017). The aim is to address the current social and health needs within the country. It is widely accepted that there is an interplay between learning and the learning environment; therefore, strategies are introduced to improve IPE activities (North et al. 2023). This initiative recognises that effective education depends on aligning both pedagogy and educational leadership with current social and health needs.

These insights from this study are critical for informing curriculum development, shaping clinical training programs and fostering a culture of collaboration within Namibia’s healthcare system. Ultimately, by equipping students with the knowledge, skills and attitudes needed for effective IPC, educational institutions can contribute to a more integrated and patient-centred healthcare system. The study aimed at exploring and describing the experiences of Faculty of Health Sciences on IPC. Understanding student’s experiences, this research aimed to contribute to the development of enhancing strong IPC and promote teamwork, effective communication and improved patient-centred care in Namibia’s healthcare system.

## Research methods and design

### Research setting

The Faculty of Health Sciences and Veterinary Medicine at the UNAM consists of various schools, namely: Pharmacy, nursing, medicine, veterinary, public health and allied health, which includes occupational therapy, physiotherapy and radiography, and is referred as to the health sciences. The Faculty of Health Sciences and Veterinary Medicine has a diverse student population comprising students from nursing, medicine, pharmacy, physiotherapy, radiography, occupational therapy and other allied health disciplines. This multidisciplinary student body provides an ideal environment for interprofessional learning and collaboration. Given its role as a health sciences faculty, the Faculty represents a microcosm of interprofessional healthcare delivery within Namibia. The complexity of services provided necessitates collaboration across professional boundaries. This setting, therefore, offered a relevant and practical environment for exploring the experiences of Faculty of Health Sciences students in IPC. Allied health sciences include radiography and occupational therapy.

### Design

The research applied a qualitative approach utilising an exploratory contextual design. Qualitative designs are best suited for uncovering insights into complex social phenomena. The exploratory approach enabled a deep understanding of Faculty of Health Sciences students’ experiences regarding IPC; the descriptive design facilitated the clear presentation of findings through coding and theming of the participants’ perspectives (Rutberg & Bouikidis 2018) and the contextual design allowed data to be collected within the participants’ experiences of IPC during their clinical placement in the health care settings, ensuring relevant and reliable insights. The approach enabled an in-depth understanding of Faculty of Health Sciences students with IPC during their clinical placement in the health care settings. Qualitative designs are best suited to uncovering insights into complex social phenomena, such as IPC, by providing rich and detailed accounts of Faculty of Health Sciences students’ experiences (Maree 2016). The study followed the Consolidated Criteria for Reporting Qualitative Research Checklist (COREQ) to guarantee comprehensive documentation of the methods used (Tong, Sainsbury & Craig 2007).

### Population and sample

The study collected data from 16 Faculty of Health Sciences students who were conveniently selected at the UNAM in Namibia. As recommended by Hirose and Creswell (2023:274), this approach targeted individuals with relevant expertise regarding the experiences of Faculty of Health Sciences students in the health care settings. These Faculty of Health Sciences students were selected as they had significant clinical exposure and experience working in multidisciplinary teams during their clinical placements.

The study employed the following eligibility criteria: (1) Faculty of Health Sciences students were either a nursing, medicine, pharmacy, radiography, occupational, dentist or physiotherapy student. (2) At least in their second year and (3) willing to participate in the study by signing an informed consent (4) available at the time of study. Data collection from Faculty of Health Sciences students proceeded until thematic saturation was achieved with 16 Faculty of Health Sciences students, defined as the point at which no new information or emergent themes were observed (Braun, Clarke & Hayfield 2022:432; Kiger & Varpio 2020:2). The criteria ensured that the study collects data from faculty of Health Sciences students who possess a significant depth of experience, enabling a more robust and insightful qualitative exploration of their experiences on IPC in the health care settings. Exclusion criteria were: First-year students, Faculty of Health Sciences students who were not willing to participate, those who agreed to participate and they were not available at the time of data collection.

### Data collection

Semi-structured interviews were used to gather data between June 2023 and September 2023. In order to investigate Faculty of Health Sciences students’ experiences with IPC, an interview guide was created based on the study’s objectives and the body of current literature. Because all Faculty of Health Sciences students were fluent in English because of their professional and academic backgrounds, the data obtained were legitimate and clear. As a professional nurse at one of the public hospitals, the main researcher had no prior personal connections with the subjects that might have affected their answers.

Participants were clearly informed of the study’s goal, the voluntary nature of participation and their right to withdraw or refuse to answer any questions at any moment in order to prevent coercion. The participants chose the dates and places for the interviews, which were usually held in peaceful areas close to their departments or in campus boardroom offices. This allowed for uninterrupted, candid conversation. The interviews were audio recorded with the participants’ consent, and field notes were obtained to record pertinent environmental observations and nonverbal clues. The duration of each interview was roughly 30 min – 45 min.

Interviews continued until no new information surfaced, which happened at the sixteenth interview. The data were considered saturated at this time. To determine the applicability and simplicity of the interview questions created, a pilot study was first carried out with four participants from the same sampling unit as the real study setting. These individuals fulfilled the same requirements as the intended audience.

The following fundamental open-ended questions were asked by the researcher, with follow-up questions based on the participants’ answers:

- *Please tell me your personal understanding of IPC?*
- *Please tell me about your experiences of IPC in health care settings among your fellow students within your faculty but from different disciplines?*
- *In your own opinion what are your recommendations or suggestions on how to improve IPC within your faculty?*

Probing questions such as ‘*What do you mean by that?*’, ‘*Could you explain further?*’ and ‘*Could you please elaborate on your suggestion?*’ were asked to elicit a deeper understanding of the phenomenon under investigation.

### Data analysis

The audio-recorded interviews were transcribed verbatim for concurrent data collection and analysis with manual coding, which involves reading over each comment and manually assigning labels. Basic demographic details of participants were collected. The researcher used an inductive approach to analyse the data, using the thematic analysis technique. The researcher used the reflexive thematic analysis approach, which focusses on people’s experiences, views, perceptions and representations of a particular phenomenon. Braun and Clarke’s six phases of thematic analysis were employed (Braun et al. 2019): Step (1) Familiarisation (getting to know the data); Step (2) Coding; Step (3) Generating themes; Step (4) Reviewing the themes; Step (5) Defining and naming themes and Step (6) Writing up the analysis and generating a report. The researcher documented his own views and previous knowledge of the phenomenon before collecting data so that he was able to engage in the self-reflective process of ‘bracketing’, whereby the researcher is expected to distinguish and set aside (but not abandon) his or her a priori knowledge and assumptions, with the goal of being open minded when listening to the participants. The author read the field notes in conjunction with the transcribed data as the data analysis process unfolded. A coding tree was designed by the first author, which highlighted the themes and described how each subtheme was developed. The coding tree also clarified which codes formed which themes. This was used to understand how the themes were created and showed that the themes came from the data as opposed to being selected beforehand. The author then resolved which themes and subthemes would be used in their reporting. An independent coder who was not one of the co-authors undertook an inquiry audit, and a consensus was reached on the themes and subthemes. This involved an analysis of the coding tree, the transcribed data and the field notes to establish that each theme was extracted from the collected data.

The independent coder had comprehensive experience in qualitative research and was also a nurse educator with a Doctorate Degree in Nursing Science.

### Trustworthiness

To ensure the credibility of the study findings, this research adhered to Lincoln and Guba’s criteria for trustworthiness: Credibility, dependability, transferability and confirmability (Lincoln & Guba 1985).

The following actions were taken during the course of the study: By ensuring a thorough data collection procedure and sustaining extended interaction with the participants for 16 weeks, credibility was developed. By clearly outlining the goal of the study and making sure participants felt at ease sharing their experiences, the researcher established trust. Member checking involved periodically verifying participant replies with each respondent to make sure they were accurate. Peer debriefing was utilised with a colleague who through regular meetings reviewed data collection, analyse coding and identify bias, ultimately ensuring study rigour and credibility. By giving a thorough explanation of the study setting, research context and participant characteristics, transferability was guaranteed. Readers can evaluate the findings’ applicability to comparable people or circumstances because of this detailed explanation. To gain a better understanding of the participants’ perspectives and experiences with IPC, verbatim quotes from them were included. By keeping an accurate and thorough audit trail of every study activity, reliability was addressed. To ensure that the research could be repeated by others, the study’s methodology, data collection instruments and analysis procedures were fully recorded. In order to ensure confirmability, the researcher actively practiced reflexivity by keeping a journal to document personal biases, assumptions and reflections throughout the study. This minimised the potential influence of the researcher’s subjective views on the findings. Additionally, all data, including audio recordings, transcripts and codes, were reviewed by a second researcher to verify the accuracy and neutrality of the interpretations. Truth value was emphasised by making sure that participant voices were accurately and respectfully represented. The pilot study did not result in any modifications to the interview guide, and the main study did not incorporate the findings from the pilot interviews.

The study collected a variety of viewpoints and experiences by enlisting a varied group of participants from several health disciplines, which helped to develop a comprehensive knowledge of IPC in the context of healthcare. The study’s conclusions are reliable and offer insightful information about health sciences students’ experiences with interprofessional teamwork in Namibia.

### Ethical considerations

An application for full ethical approval was made to the University of Namibia’s Research Ethics Committee on 30 October 2020. The ethics approval number is SoNEC 53/2020 and the Ministry of Health and Social Services research unit on 08 December 2020. The ethical clearance number is RSM 2020. The study adhered to ethical guidelines to protect participants’ rights and ensure the integrity of the research process. The study adhered to ethical guidelines to protect participants’ rights and ensure the integrity of the research process.

The Executive Dean of the UNAM’s Faculty of Health Sciences and Veterinary Medicine office gave the researcher permission to carry out the study after relevant permissions were received and helped to facilitate communication with participants. Participation was voluntary, and informed consent was obtained from all interviewees. Complete confidentiality was not guaranteed because the researchers could not control what the educators would talk about after the interviews as the researchers knew their interviewees. The goal of the study, its methods and participants’ rights, including the ability to withdraw at any moment, were all thoroughly explained to the participants. Before the interviews started, each participant provided written informed consent. Participants were given pseudonyms, and all data were safely stored to ensure confidentiality. Data were safely stored for 5 years at the conclusion of the study before being deleted in compliance with institutional guidelines.

## Results

### Participants’ characteristics

A total of 16 participants from the Faculty of Health Sciences at the UNAM took part in this study. The participants were drawn from various health disciplines, including nursing (4), medicine (3), pharmacy (3), dentistry (1), physiotherapy (1), occupational therapy (1) and radiography (3). All participants had completed at least 1 year of clinical placement, ensuring they had firsthand exposure to IPC in healthcare settings. The participants’ ages ranged from 21 to 33 years, with the majority (11 participants) between the ages of 21 and 25 years. Gender distribution included 10 females and 6 males, reflecting the composition of health sciences students at UNAM. In terms of year of study, most participants were in their third (five participants) or fourth (six participants) year of training, while the remaining three were in their second or fifth year of study. This diverse sample allowed for the exploration of different perspectives on IPC, ensuring a well-rounded understanding of how students from various disciplines engage in collaborative practices

Table 1 presents the demographic profile of the 16 participants included in the study. The participants were drawn from various health science disciplines and different years of study, with both male and female students represented. This diversity allowed for a comprehensive exploration of students’ experiences of IPC in healthcare settings.

**TABLE 1:** Demographic characteristics of participants (*N* = 16).

Participant	Age (years)	Gender	Discipline	Year of study
P1	22	Female	Nursing	Third
P2	24	Female	Nursing	Fourth
P3	23	Female	Pharmacy	Fourth
P4	25	Male	Nursing	Fifth
P5	26	Male	Medicine	Fourth
P6	22	Male	Nursing	Third
P7	24	Female	Nursing	Fourth
P8	27	Female	Medicine	Fifth
P9	23	Female	Radiography	Third
P10	21	Female	Pharmacy	Second
P11	25	Female	Pharmacy	Fourth
P12	28	Female	Occupational Therapy	Fifth
P13	22	Female	Radiography	Third
P14	31	Female	Nursing	Fourth
P15	33	Male	Dentistry	Fifth
P16	30	Male	Physiotherapy	Second

### Participants experiences of interprofessional collaboration in the healthcare settings

The data analysis revealed three major themes, each containing multiple subthemes that reflect participant’s experiences on IPC, the barriers they encountered and their recommendations for improving interprofessional engagement. The thematic analysis identified three main themes: (1) Participants’ understanding of IPC, (2) barriers to effective IPC in clinical settings (3) mechanisms for enhancing IPC in healthcare settings.

The thematic analysis of the interview data yielded three main themes and several associated subthemes, as presented in Table 2, reflecting participants’ understanding of IPC, barriers to effective collaboration and strategies for enhancing IPC in healthcare settings.

**TABLE 2:** Themes and subthemes.

Themes	Subthemes
Theme 1: Participants’ understanding of interprofessional collaboration.	1. Value of teamwork and patient-centred care 2. Learning from other disciplines through IPC 3. Enhancing clinical decision-making through IPC 4. Nursing care plan or patient management. 5. Ways of improving services
Theme 2: Barriers to effective IPC in clinical settings	1. Lack of understanding of roles within IPC 2. Hierarchical dynamics in clinical settings 3. Poor communication among disciplines 4. Limited exposure to interprofessional engagement
Theme 3: Mechanisms for enhancing IPC in healthcare settings	1. A module on IPC to be introduced prior to clinical placement 2. Improve interprofessional relationships through open communication 3. Establishing joint interprofessional ward rounds 4. Encouraging mentorship and leadership for IPC 5. Strengthening institutional support for IPC implementation

IPC, interprofessional collaboration.

### Theme 1: Participants’ understanding of interprofessional collaboration

This theme highlights how students perceive IPC as an essential aspect of healthcare training, allowing them to develop a deeper understanding of teamwork, professional roles and patient-centred care. Participants viewed IPC as an opportunity to enhance their clinical knowledge, improve communication skills and work effectively within interdisciplinary teams.

#### Subtheme 1: Value of teamwork and patient-centred care

Participants emphasised that working in interdisciplinary teams improved patient care and service delivery. Many believed that collaboration among health professionals ensures holistic patient management, reducing the risk of fragmented care.

One participant stated:

‘Interprofessional collaboration allows us to work towards a common goal – better patient care. When we understand each other’s roles, we communicate better and make decisions as a team.’ (P3, Female, Pharmacy)

Another participant noted:

‘When doctors, nurses, and pharmacists work together, we can develop the best treatment plan for patients. It reduces medical errors and ensures all aspects of the patient’s needs are met.’ (P7, Female, Nursing)

#### Subtheme 2: Learning from other disciplines through interprofessional collaboration

Participants reported that IPC provided them with opportunities to learn from other disciplines, broadening their understanding of different healthcare roles and responsibilities.

One participant shared:

‘Before working with other students in a clinical setting, I didn’t really know what a physiotherapist did. But seeing them help patients regain mobility showed me how important their role is.’ (P5, Male, Medicine)

#### Subtheme 3: Enhancing clinical decision-making through interprofessional collaboration

Participants noted that collaborating with professionals from different disciplines enhances problem-solving and clinical decision-making.

One participant stated:

‘During ward rounds, we discussed cases with medical students, nurses, and pharmacists. Each person contributed a different perspective, and it really helped in making well-informed decisions.’ (P8, Female, Medicine)

#### Subtheme 4: Nursing care plan or patient management

Participants highlighted the importance of nursing care plans in ensuring comprehensive and well-coordinated patient management. They emphasised that through IPC, healthcare providers could develop individualised care plans that address all aspects of a patient’s health.

One participant shared:

‘As a nurse, I rely on input from other professionals, such as doctors and physiotherapists, to create a care plan that best supports the patient’s recovery process.’ (P14, Female, Nursing)

Another participant added:

‘When we discuss a patient’s progress in a team, it helps us refine the care plan and ensure the best possible outcomes. Every professional has something valuable to contribute.’ (P16, Male, Physiotherapy)

#### Subtheme 5: Ways of improving services

Participants emphasised that IPC plays a crucial role in improving healthcare services by fostering better communication, enhancing efficiency and reducing redundancies in patient care. They noted that working collaboratively allows professionals to identify gaps in service delivery and implement solutions that benefit both healthcare providers and patients.

One participant stated:

‘Interprofessional collaboration helps us streamline services. When we communicate effectively, we eliminate unnecessary delays and improve patient care.’ (P16, Male, Physiotherapy)

### Theme 2: Barriers to effective interprofessional collaboration in clinical settings

Despite recognising the benefits of IPC, participants reported several challenges that hindered effective collaboration. These barriers included lack of role clarity, hierarchical structures, poor communication and limited exposure to IPC practices.

#### Subtheme 1: Lack of understanding of roles within interprofessional collaboration

Many participants struggled to understand the roles and responsibilities of other disciplines, making collaboration difficult. Role ambiguity often led to confusion in decision-making, delays in patient care and miscommunication among healthcare professionals.

One participant stated:

‘Sometimes I wasn’t sure when to consult a pharmacist about a patient’s medication or whether I should only rely on the doctor’s prescription.’ (P9, Female, Radiography)

Another participant noted:

‘Without clear guidelines, it’s hard to know who is responsible for what. It leads to confusion and delays in patient care.’ (P6, Male, Nursing)

#### Subtheme 2: Hierarchical dynamics in clinical settings

Several participants expressed frustration with hierarchical structures, in which some professions were given more authority than others, making it difficult for students and other healthcare workers to engage in decision-making. Many noted that strict hierarchies discouraged open dialogue and collaboration.

One participant stated:

‘In many cases, doctors are the only ones making decisions. Other professionals, including students, are expected to follow orders rather than contribute.’ (P11, Female, Pharmacy)

Another participant added:

‘There’s a culture where junior staff and certain professions feel unheard in decision-making. This limits our ability to work effectively as a team.’ (P15, Male, Dentistry)

#### Subtheme 3: Poor communication among disciplines

Participants emphasised that ineffective communication led to misunderstandings and disrupted teamwork, often resulting in fragmented patient care and clinical errors. Clear and timely communication is a fundamental component of IPC, as it ensures that healthcare teams operate cohesively and make informed decisions.

One participant shared:

‘There were times when key patient information wasn’t communicated properly, leading to confusion and errors.’ (P10, Female, Pharmacy)

Participants noted that there were few opportunities for structured interprofessional learning and collaboration in their training. Many reported that students from different disciplines interacted minimally unless required for a specific case.

One participant shared:

‘Most of the time, we stayed within our own professional groups. We didn’t really get to work with other students unless it was necessary, which limited how much we learned about IPC.’ (P13, Female, Radiography)

#### Subtheme 4: Limited exposure to interprofessional engagement

Participants reported that limited exposure to structured IPC opportunities during their clinical placements hindered their ability to develop effective teamwork and communication skills. Many students noted that their training primarily occurred within their respective disciplines, with minimal interaction with students or professionals from other healthcare fields.

One participant expressed concern over this issue:

‘Most of the time, we stayed within our own professional groups. We didn’t really get to work with other students unless it was necessary, which limited how much we learned about IPC.’ (P13, Female, Radiography)

### Theme 3: Mechanisms for enhancing interprofessional collaboration in healthcare setting

This theme highlights key strategies suggested by participants to enhance IPC in healthcare settings. Participants emphasised the importance of structured training, improved communication and joint ward rounds to strengthen interdisciplinary teamwork and improve patient care.

#### Subtheme 1: A module on interprofessional collaboration to be introduced prior to clinical placement

Participants emphasised the need for structured IPC training before students begin their clinical placements. Many expressed that early exposure to IPC principles would better prepare them for real-world collaboration in healthcare settings.

One participant shared:

‘A structured IPC module would help eliminate confusion about roles and responsibilities in clinical settings. It would make collaboration much smoother.’ (P3, Female, Pharmacy)

#### Subtheme 2: Improving interprofessional relationships through open communication

Participants identified communication as a critical factor in effective IPC. They noted that strengthening open communication between healthcare professionals would lead to better collaboration and improved patient care.

One participant stated:

‘Effective communication builds trust among professionals, making it easier to work together towards patient-centered care.’ (P2, Female, Nursing)

Another participant added:

‘Lack of communication creates unnecessary conflicts and delays. Encouraging open discussions can improve collaboration significantly.’ (P9, Female, Radiography)

#### Subtheme 3: Establishing joint interprofessional ward rounds

Participants emphasised that joint interprofessional ward rounds would maximise the use of interdisciplinary expertise, allowing for better patient management and teamwork among professionals.

One participant shared:

‘Having different professionals participate in ward rounds means we can make better-informed decisions for patients, rather than working in silos.’ (P10, Female, Pharmacy)

#### Subtheme 4: Encouraging mentorship and leadership for interprofessional collaboration

Participants suggested that mentors should guide students in IPC and demonstrate effective teamwork. They emphasised that experienced professionals could help shape students’ attitudes towards collaboration by modelling best practices in interprofessional engagement.

One participant suggested:

‘If our supervisors encouraged interprofessional teamwork instead of keeping us in silos, we would feel more comfortable working with other professionals.’ (P12, Female, Occupational Therapy)

#### Subtheme 5: Strengthening institutional support for interprofessional collaboration implementation

Participants emphasised that the successful integration of IPC into healthcare training requires institutional commitment from universities, hospitals and healthcare policymakers. They felt that institutions must actively promote and support IPC through policies, curricula and resource allocation.

One participant suggested:

‘If IPC was prioritised by both the university and hospital administration, we would have more opportunities to collaborate and learn from each other before entering the workforce.’ (P2, Female, Nursing)

## Discussion

The purpose of this study was to explore and describe the experiences of Faculty of Health Sciences students regarding IPC in healthcare settings within the Khomas Region, Namibia.

The findings revealed that while students generally recognised IPC as vital to improving patient care and professional growth, they encountered significant barriers that limited effective collaboration. Similarly, studies conducted in both high- and low-resource contexts confirm that health professionals conceptualise IPC as essential to addressing complex patient needs (Orchard, Curran & Kabene 2022; Reeves, Palaganas & Zierler 2017).

The study found that students viewed IPC as an essential component of quality healthcare delivery and professional development. Participants highlighted teamwork, shared learning and patient-centred care as key benefits of working collaboratively with peers from other health disciplines. These findings are consistent with previous studies that emphasise IPC’s role in fostering mutual respect, improving communication and enhancing patient outcomes (Reeves et al. 2010; Schot et al. 2020). Students’ descriptions of learning from other disciplines mirror the findings of Slaghmuylder et al. (2025), who argue that IPC builds appreciation for the distinct but complementary roles within a healthcare team. Overall, Martimianakis et al. (2020) found that collaboration, when role-model during problem-solving, creates conditions for understanding ‘why’ collaboration matters, while Paxino et al. (2024) demonstrated that team meetings provide practical, time-efficient learning opportunities.

Participants reported that IPC enriched their clinical decision-making by exposing them to diverse professional perspectives, which aligns with findings by Botma and Snyman (2019), who highlight that multidisciplinary collaboration improves diagnostic accuracy and decision quality. The emphasis students placed on teamwork and patient-centred care also reflects the core competencies of IPC outlined by Mohammed, Anand and Saleena Ummer (2021), namely, values and ethics, roles and responsibilities, communication and teamwork. This finding aligns with Katantha et al. (2025) who emphasise that incomplete or one-way communication compromises patient safety, diminishes trust among team members and reduces collaboration efficiency. International evidence mirrors this, with studies in Kenya demonstrating similar challenges in which written-only orders delayed care and hindered collaboration between nurses and doctors (Nzinga, McGivern & English 2019).

These competencies are foundational for preparing students for collaborative clinical practice. However, Zhao et al. (2024) study that collectively represented observations from approximately 200–300 healthcare professionals across multiple settings, providing a moderate-strength evidence base for IPC’s learning potential, cautioned that empirical evidence for individual learning remains relatively weak, suggesting the need for more rigorous research.

Despite these positive perceptions, several barriers were identified that hindered the effective practice of IPC.

The most notable challenges included role ambiguity, hierarchical structures, poor communication and limited exposure to structured interprofessional engagement. Research further suggests that fostering a shared interprofessional identity can attenuate hierarchical influence, encouraging more equitable participation across professional boundaries (Schot et al. 2020). These barriers have also been documented in prior studies (Smets & Visser 2023). The evidence suggests these barriers are widespread and require comprehensive, multi-level interventions to overcome.

Participants’ accounts of uncertainty regarding professional roles illustrate a lack of clarity in defining the scope of practice across disciplines, leading to confusion and duplication of tasks. This supports findings by Oandasan and Reeves (2005), who assert that unclear professional boundaries weaken collaboration and compromise patient care. Consequently, Jabbar et al. (2023) specifically highlighted that 68.6% of healthcare professionals reported role and leadership ambiguity as a significant barrier, and 68.1% noted different individual team members’ goals. Overall, Rawlinson et al. (2021) identified unclear roles as one of the main obstacles; however, they also found that at times, lack of time and fears about professional identity may also hindered the effective practice of IPC.

Hierarchical dynamics were another major obstacle. Students reported that decision-making often remained dominated by certain professions, particularly medicine, which discouraged open dialogue and collaboration.

Similar findings by O’Rourke et al. (eds. 2013) and Reeves et al. (2010) indicate that power imbalances undermine team cohesion and restrict knowledge sharing. In the Namibian context, such hierarchies may be further entrenched by traditional healthcare models that prioritise physician authority over interdisciplinary consensus.

Poor communication also emerged as a critical barrier. Participants recounted instances of miscommunication and inadequate information sharing among team members. This aligns with the work of Abd Hamid et al. (2016), who found that communication breakdowns significantly contribute to medical errors and inefficiencies. Strengthening structured communication channels, such as regular multidisciplinary meetings or electronic health record systems, could help mitigate these issues.

Finally, limited exposure to IPC during clinical placements restricted students’ opportunities to develop collaborative competencies. This finding is consistent with El-Awaisi et al. (2024), who warn that insufficient exposure to interdisciplinary practice during training can reinforce professional silos and hinder the development of teamwork skills. Therefore, the systematic integration of IPC into the curriculum is essential to equip students with the necessary skills for future collaborative practice.

Participants proposed several strategies to strengthen IPC within healthcare training and practice. These included introducing a structured IPC module before clinical placements, promoting open communication, establishing joint ward rounds, encouraging mentorship and reinforcing institutional support. Such recommendations are consistent with global best practices in IPE and collaboration (Barr et al. 2016; Freeth et al. 2005).

The suggestion to introduce a pre-clinical IPC module is particularly noteworthy. As Oandasan and Reeves (2005) argue, early exposure to collaborative principles fosters an understanding of teamwork and prepares students for interdisciplinary environments. Mentorship and leadership were also emphasised as critical to nurturing positive attitudes towards IPC, echoing findings by Freeth et al. (2005), who identified mentoring as a catalyst for sustained collaboration among healthcare students.

Furthermore, critically, successful collaboration requires systemic interventions addressing individual, interactional, professional and organisational dimensions (Dib & Belrhiti 2025). Therefore, participants’ call for institutional support underscores the importance of organisational commitment in embedding IPC within training and healthcare systems. Institutions that prioritise IPC through curriculum design, policy development and interdepartmental collaboration are more likely to produce graduates who are competent in teamwork and communication (Nash 2023; Reeves et al. 2010). In the Namibian context, this may involve universities and the Ministry of Health aligning efforts to ensure that clinical education environments actively promote interdisciplinary collaboration.

### Limitations, strengths and recommendations

Interprofessional collaboration is integral to enhancing healthcare delivery; however, this study presents certain limitations that should be acknowledged. One of the key limitations is the study’s scope, as it was conducted within a specific healthcare setting, potentially restricting the transferability of the findings to other regions or healthcare systems. Furthermore, reliance on self-reported data introduces the possibility of recall bias or social desirability bias, which may have influenced participants’ responses limits the breadth of perspectives captured, which could affect the overall representation of interprofessional experiences across diverse healthcare disciplines. Participants were recruited through convenience sampling, in which the representation of across the school varies. This may suggest that there was or there may have been volunteer bias. Despite these limitations, the study offers significant contributions to the understanding of IPC in healthcare education and practice. Employing a qualitative approach, the research provides an in-depth exploration of students’ experiences, uncovering critical challenges and opportunities for enhancing collaboration in multidisciplinary healthcare teams. Future research should focus on expanding the scope of IPC studies across various healthcare settings, including rural and resource-limited environments, to provide a more comprehensive understanding of the barriers and facilitators of effective collaboration. Additionally, employing mixed-method approaches with larger sample sizes could improve the generalisability of findings. Longitudinal studies examining the long-term impact of Interprofessional education and training on healthcare delivery and patient outcomes would also offer valuable insights for informing policy development and curricular reforms aimed at fostering a collaborative healthcare workforce.

## Conclusion

The study highlights that while students recognise the importance of IPC, they face significant barriers that hinder its implementation. Lack of role clarity, hierarchical structures and poor communication were identified as key challenges. However, students also proposed solutions, such as structured IPC training, mentorship and better institutional support to improve collaborative healthcare delivery. By addressing these challenges and implementing these recommendations, universities and healthcare institutions can foster a culture of IPC that benefits both students and patients. The study also contributes to the limited body of literature on IPC in Namibia, providing valuable evidence that can inform curriculum development, clinical training and healthcare policies. Additionally, the use of semi-structured interviews and thematic analysis allowed for a rich understanding of students’ perspectives, making the findings highly relevant for education and practice.
